# Mid-term results of intra-articular calcaneal fractures treated with minimally invasive two-point distractor: Single center study with 6-year follow-up

**DOI:** 10.1007/s00508-024-02476-5

**Published:** 2024-12-02

**Authors:** Matthias Stark, Domenik Popp, Lukas Schmoelz, Kevin Doering, Kerstin Stark, Arastoo Nia

**Affiliations:** 1 Department of Orthopedic Surgery, Krankenhaus Neunkirchen, Danube Private University, Neunkirchen, Niederösterreich Austria; 2 Department of Orthopedics and Trauma Surgery, Division of Trauma Surgery, Medical University of Vienna, Währinger Straße 18–20, 1090 Vienna, Austria; 3 Department of Orthopedic and Trauma Surgery, Krankenhaus Wiener Neustadt, Wiener Neustadt, Niederösterreich Austria

**Keywords:** Displaced calcaneal fracture, Minimally invasive surgery, Functional outcome, Fröhlich Distractor, ORIF

## Abstract

**Background:**

Displaced intra-articular calcaneal fractures are a complication-ridden injury to treat and there are various treatment techniques to address this injury. The aim of this study was to evaluate the mid-term outcome of a percutaneous two-point distractor technique in patients with displaced intra-articular calcaneal fractures.

**Methods:**

A retrospective data analysis of patients with intra-articular calcaneal fractures treated in a level 1 trauma center was conducted. The patients were subsequently invited for a follow-up visit to assess the mid-term outcome. The Böhler’s and Gissane’s angles, the American Orthopaedic Foot & Ankle Society hindfoot score, the Maryland foot score and a visual analog scale for pain were measured preoperatively and postoperatively and after at least 6 years to assess the radiological and functional outcome.

**Results:**

Of the patients 59 completed the study with a mean follow-up of 76 months after surgery. The mean American Orthopedic Foot and Ankle Society hindfoot score at the last follow-up visit was 81, the mean Maryland foot score was 78. There were three cases (5%) of superficial wound complications and secondary arthrodesis of the subtalar joint was performed in five patients (8%).

**Conclusion:**

The low rates of postoperative infections and secondary arthrodesis in this study underline the good to excellent mid-term functional results for a minimally invasive technique.

## Introduction

Displaced intra-articular calcaneal fractures (DIACF) are of particular concern to trauma surgeons because even small displacements and subsequent incongruencies mainly in the subtalar joint are associated with a poor long-term outcome. Due to the complex anatomy of the subtalar joint complex and the hindfoot, serious impairments with the risk of permanent disability as a result of the fracture are common [1]. Anatomical reduction of the posterior calcaneal facet has been the gold standard for the surgical treatment of calcaneal fractures for decades [2]. The widely utilized open reduction and internal fixation (ORIF) procedure uses a lateral approach with internal plate fixation and provides full visibility to optimize the positioning of fracture parts [3]. The significant shortcomings of this approach are a usual postponement of surgery until a sufficient reduction in swelling is reached and a relatively high number of wound healing problems, which is around 15–37% [3].

Therefore, various minimally invasive approaches have been proposed to minimize soft tissue damage and to reduce wound healing complications [4]. A significant issue of minimally invasive approaches is a satisfactory anatomical alignment of the calcaneus, which can be harder to achieve in comparison to open techniques. The surgical method described by Fröhlich et al. [5] uses an external 2‑point distractor that leads to a satisfactory realignment of the fracture fragments and does not interfere with radiological imaging during surgery. This approach allows a precise placement of percutaneous screws for stabilizing the fragments and showed a low complication rate in the literature [5, 6]; however, mid-term follow-up data of patients treated with this technique are sparse. Thus, this study aims to analyze mid-term outcomes of types II, III and IV displaced calcaneal fractures according to the Sanders classification and treated with a minimally invasive approach [7].

## Methods

All patients who initially presented at our level 1 trauma center at Landeskrankenhaus Wiener Neustadt in Vienna with type II, III or IV calcaneal fractures according to the Sanders classification and who were treated by minimally invasive surgery according to Fröhlich et al. between January 2009 and December 2018, met the inclusion criteria and were identified, invited and examined after providing signed informed consent.

Demographic data, cause of injury, treatment modality and time to surgery were collected using our institution’s available patient files. In addition, all calcaneal fractures were classified based on the severity according to the Sanders classification. Preoperative and postoperative measurements of the Böhler’s and Gissane’s angle were obtained and compared with the measurements on the follow-up radiographs. The anterior posterior, lateral and axial radiographs were classified and evaluated by one author.

Complications such as postoperative wound infections and the need for a subtalar arthrodesis were recorded. Functional and clinical outcomes were evaluated with the American Orthopaedic Foot & Ankle Society (AOFAS) hindfoot score, the Maryland foot score (MFS) and a visual analog scale (VAS) for participant pain. For the evaluation the MFS was divided into four categories: a score of ≥ 9 was considered excellent, a score of < 9 and ≥ 8 was considered good, a score of < 8 and ≥ 6 was considered fair and a score of < 6 was considered poor.

Surgery was performed by orthopedic and trauma surgeons who were skilled in ORIF and minimally invasive techniques.

## Surgical technique

**Fig. 1:**
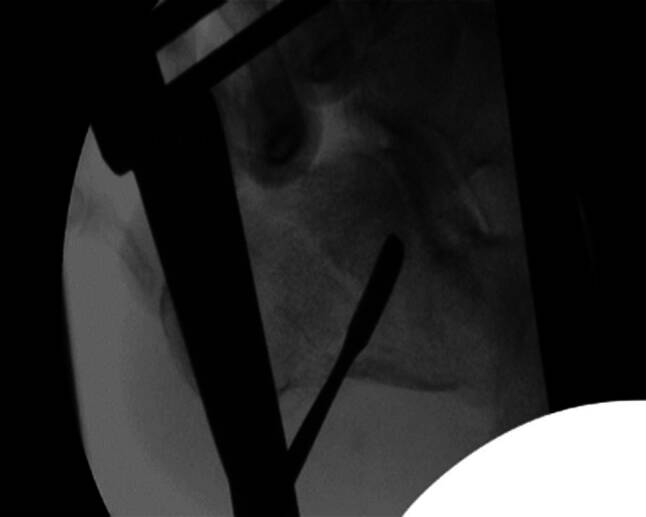
Broden View of the posterior joint facet

Early surgery was performed if the tissue conditions were adequate. The patient was placed in a lateral position with the fractured side up, close to the edge of the operating table. Standard lateral and Broden view radiographs were obtained. A 3 mm K-wire was positioned perpendicular to the subtalar joint on the lateral view and two 3 mm K-wires were separately placed perpendicularly to the calcaneal axis in the talus neck and into the calcaneal tuberosity. Subsequently, the Fröhlich traction device (ITS Implants, Autal, Laßnitzhöhe, Austria) was assembled on both sides of the calcaneus and traction was gradually initiated. The subtalar joint traction was monitored by lateral view. Through a lateral incision, a raspatory or drill wire is used to lift and reduce the dislocated and impressed parts of the posterior joint facet (Fig. 1).

**Fig. 2:**
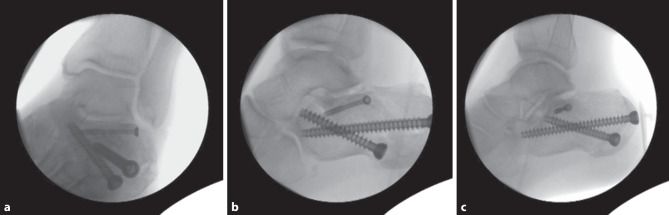
Lateral view of the calcaneus fracture treated with closed reduction percutaneous fixation surgery showing (**a**) oblique view showing thew sustentaculum screw and two 7.3 mm diameter self-tapping screws, (**b**) view of the intraoperative reduction and (**c**) postoperative final reduction

The length of the calcaneus was reduced, and the subtalar joint line was viewed to elevate the compressed articular surface and lateral wall. Then, an axial view of the calcaneus was obtained to permit calcaneal varus and valgus adjustments by retracting the unilateral or bilateral traction device. The lateral and medial surface of the subtalar joint can be reduced and temporarily fixed using a 4.0-mm cannulated screw guide wire percutaneously. Subtalar joint reductions were completed and assessed by lateral and axial views. The sustentaculum screw was then placed from lateral dorsal to medial ventral below and dorsal to the outer ankle tip, if the reposition was appropriate using a 4.0-mm cannulated screw for subtalar articular fixation. A second screw was additionally positioned if required. Then the calcaneus was screwed longitudinally with cancellous bone screws with a continuous thread lengthways from the tuber to the anterior process and also obliquely from the tuberosity lateral to the medial sustentaculum tali fragment with two 7.3 mm diameter self-tapping screws with a continuous thread, which is essential for maintaining the length and axis of the calcaneus (Fig. 2).

**Fig. 3:**
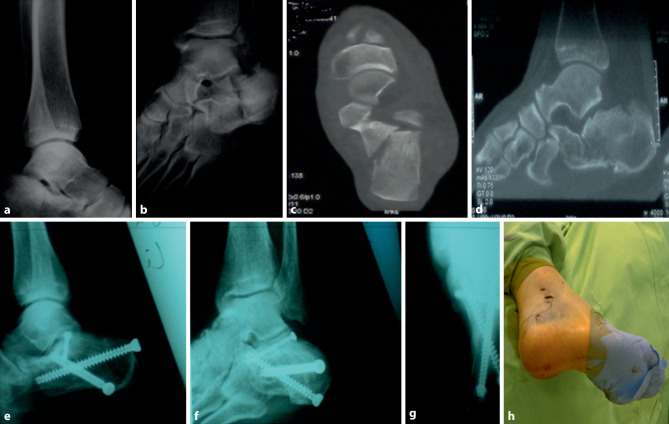
Patient case. Patient with intra-articular calcaneal fracture after fall from a great height. Sanders IIA type fracture, treated with 2‑point distractor fixation technique. **a** Preoperative lateral X‑ray and **b** preoperative axial X‑ray. Computed tomography (CT) image **c**, **d**). **e** Postoperative lateral X‑ray shows good reconstruction of Böhler’s angle and Gissane’s angle. **f** Postoperative oblique X‑ray shows good reduction of calcaneal height. **g** Postoperative axial X‑ray shows good reduction of calcaneal length. **h** Intraoperative incision picture

Finally, the distractor was removed and the stab incisions closed with simple sutures. An elastic bandage is applied postoperatively and the injured foot is kept non-weight bearing for 8 weeks. Toe and ankle range of motion exercises can be started immediately after the swelling has subsided. A patient case is displayed in Fig. 3.

### Ethical assessment

Informed consent to participate was obtained from all individual patients included in the study. The local ethics committee approved the study protocol.

The gathered data were analyzed using the SPSS statistical software version 23 (SPSS Inc., Chicago, IL, USA). Radiologic parameters at each time point were compared using repeated measures ANOVA. The Mann-Whitney test was used to compare patient groups according to clinical outcomes. Statistical significance was defined as *P* < 0.05.

## Results

**Fig. 4:**
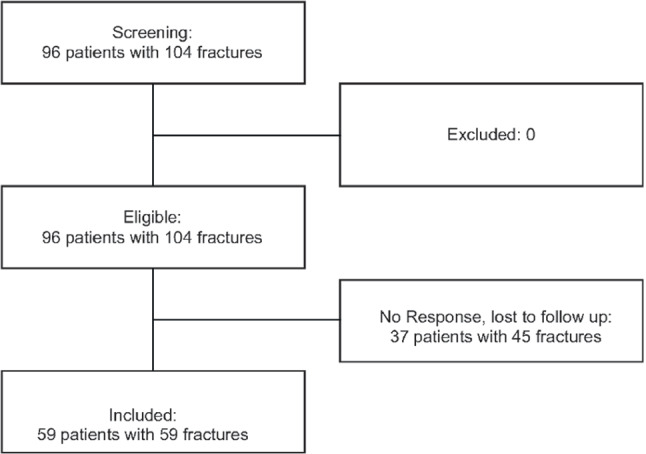
Flowchart showing the process of inclusion of patients in the study

Between 2009 and 2018 a total of 96 patients with intra-articular calcaneal fractures were treated operatively and 37 were excluded from this study due to lost to follow-up. In total, 59 patients were included, 51 patients were male and 8 were female. The mean age was 49 years (range 23–60 years) (Fig. 4).

All 59 patients had a unilateral fracture. According to the Sanders classification 11 patients (18%) were diagnosed with a type 2 fracture, 32 (55%) type 3 and 16 (27%) were classified as a type 4 fracture.

The mean time to surgery was 4.5 days. Overall hospital stay was 6.9 days, 7 patients (11.8%) stated that they were active smokers and 3 (5%) were diagnosed with diabetes mellitus before surgery (Table 1). The mean time for follow-up was 76 months (range 24–132 months).

**Table 1:** Demography

Value	*N (percentage)*
*Age (years)*	49 (range 23–60)
*Gender*	
Male	51 (86.4%)
Female	8 (13.6%)
*Sanders type*	
Type 2	11 (18%)
Type 3	32 (55%)
Type 4	16 (27%)
*Fracture site*	
Unilateral	59
*Cause of Trauma*	
Fall from a height	52 (88.1%)
Traffic accident	7 (11.9%)
*Clinical information*	
Diabetes	3
Smoker	7
Time until surgery	4.5 ± 3.7 days
Length of hospital stay	6.9 ± 2.8 days
*Postoperative complications*	
Arthrodesis	5 (8%)
Superficial infection	3 (5%)

The results of the radiographic examination of the investigated feet at three different points in time are presented in Table 2.

**Table 2:** Degrees (°) of Böhler’s and Gisanne’s angles at three time points

Radiographic parameters	Mean (*n* = 59)	Sanders II (*n* = 11)	*p*-value	Sanders III (*n* = 32)	*P*-value	Sander IV (*n* = 16)	*p*-value
Böhler’s angle before surgery	24	27.2 (range 15.1–36.7)	–	26.2 (range 15.0–34.5)	–	12.3 (range 1.3–30.6)	–
Böhler’s angle after surgery	30	30.8 (range 30.8–39.6)	< 0.001	28.2 (range 24.8–39.3)	< 0.001	27.1 (range 18.7–38.5)	< 0.001
Böhler’s angle at follow-up	28	29.6 (range 24.3–38.9)	0.023	28.0 (range 23.3–35.7)	0.01	26.2 (range 20–36.4)	0.008
Gissane’s angle before surgery	108	112.4 (range 91.4–125.0)	–	109.5 (range 89.7–122.1)	–	106.3 (range 20.0–130.8)	–
Gissane’s angle after surgery	115	117.4 (range 102.4–128.7)	0.19	109.4 (range 100.4–121.6)	0.008	118.2 (range 105.2–128.7)	< 0.001
Gissane’s angle at follow-up	114	116.3 (range 102.5–127.9)	1.000	108.3 (range 96.9–121.0)	1.000	116.1 (range 95.3–127.1)	1.000

There were significant differences between the preoperative and postoperative and between the postoperative and last follow-up especially for Böhlers angle, while there was no significant difference between the other periods. Of the patients five required a subtalar arthrodesis (8%). The overall rate of complications was 5% due to superficial infection (*n* = 3). In 33 cases there was an elective implant removal with a mean time to implant removal of 21 weeks (range 11–30 weeks). Functional outcomes at follow-up are shown in Table 3.

**Table 3:** Functional outcome according to AOFAS and MFS and VAS

Clinical and functional outcome score	Mean	Excellent	Good	Fair	Poor
*AOFAS*	81	–	–	–	–
Sanders II	87	4	4	3	0
Sanders III	80	15	10	5	2
Sanders IV	75	2	4	2	8
*MFS*	78	–	–	–	–
Sanders II	85	5	5	1	0
Sanders III	79	5	15	8	4
Sanders IV	70	0	4	8	4
*VAS*	3.1	–	–	–	–
Sanders II	2.3	7	4	0	0
Sanders III	3.5	8	20	3	1
Sanders IV	3.2	4	1	11	0

*AOFAS* American Orthopaedic Foot and Ankle Society, *MFS* Maryland foot score, *VAS* Visual analog scale

Regarding pain, the patients with Sanders types III and IV fractures had an average VAS score for pain of 3.5 and 3.2, respectively. Those with type 2 fractures had lower average scores of 2.3 points.

Concerning mobility 35 (60%) patients were able to wear normal shoes in everyday life, 24 (40%) required insoles or orthopedic footwear, 43 patients (72.9%) were able to walk more than 1 km without any problems, 13 patients (22%) up to 1 km and 3 patients (5.1%) were severely restricted with a walking distance of less than 100 m.

## Discussion

The primary focus of this study was to assess the postoperative midterm outcomes for patients with DIACF treated with a 2-point distractor method.

The current treatment of choice for displaced intra-articular fractures of the calcaneus is controversial. Although ORIF has the possibility of anatomical reduction, its main disadvantages remain as wound complications and infections are reported in up to 30% of patients [8]. To reduce these complications, many techniques for minimally invasive reduction and fixation of DIACF have been developed. In recent years, percutaneous fixation, external fixation, arthroscopically assisted fixation and minimal incision techniques have been described in the literature [9]. Percutaneous reduction and fixation with cannulated screws has therefore gained more popularity in the treatment of calcaneal fractures. Due to the advantages of minimally invasive surgery, they offer less morbidity, earlier recovery, a potentially decreased rehabilitation period and less tissue traumatization [10].

Most cases of calcaneal fractures are associated with severe swelling after injury, and an early operative intervention of intra-articular calcaneal fractures is extremely crucial [11]. To achieve the best results, timing is an important factor and surgery should be done within 5 days, especially percutaneous or minimally invasive surgery [12]. Percutaneous fixation techniques can be done within a short time after injury. Rodemund et al. [6]described a time to surgery of 2.7 days, which is even faster than our results of 4.5 days.

The AOFAS score provides a widely used tool for evaluating functional outcomes in patients with foot injuries. The reported AOFAS hindfoot scores for minimally invasive procedures are around 76–83 [13]. A probable explanation for the general heterogeneity in the literature is the different types of fractures and the different times of follow-up [14].

We reported a mean score for AOFAS of 81 and for MFS of 78 for follow-up, which were almost equal if compared to studies with comparable surgical techniques. Tomesen et al. achieved mean values of the AOFAS and MFS scores 84.1 and 86.4 points, respectively, with a mean follow-up of 66 months [15]. Schepers et al. used the technique described by Forgon in 61 calcaneal fractures. In that study, the mean values of the AOFAS and MFS scores were 83 and 79 points, respectively [16].

Evaluation of the outcome of DIACF can be challenging because of the many different available methods. A relatively common approach is using radiological measurements, such as the Böhler angle and the Gissane angle. These two parameters allow a relatively fast and easy evaluation of the anatomical position of the calcaneus [17].

The postoperative Böhler angle for patients treated with ORIF is described as around 26° [18, 19]. We achieved mean results between 27° and 30°, which is in line with other minimally invasive procedures, which were between 26° and 28° [12, 14]. At the follow-up time, a reduction of around 4–6° seems to be common [19, 20]. Our results are slightly lower with around 2°. The reason might be due to an increase of axial loading as once the patient starts with weight bearing the Achilles tendon and the foot flexors pull the calcaneus towards the talus [19]. A similar outcome can be observed for the Gissane angle [21].

Infections after calcaneal fractures are a problem. A significant advantage of a minimally invasive technique is the comparably lesser risk of postoperative infections and soft tissue damage. The main purpose of fixation with an external device is to provide sufficient reduction while avoiding the exposure of soft tissue [22]. The ORIF procedures within the first week post-injury lead to an increased incidence of wound healing problems [23]. Backes et al. reported an infection rate for ORIF of 2–25% of patients treated with the ORIF approach, with a 21% chance for further surgical revision [24]. The prospective randomized multicenter study of Buckley et al. in 2002 with over 300 DIACFs showed a superficial infection and wound complication rate of 17% for ORIF [25].

Infections with the minimally invasive approach are described to be between 3% and 18% [13, 26]. Our collective showed an infection rate of 5%, which is comparable to other minimally invasive techniques. It should be noted that these were superficial wound infections.

In our study 5 patients (8%) required secondary subtalar arthrodesis, which is slightly higher than the reported rate of the literature with 2.1–7.3% [11, 19] and less than in non-surgically treated patients. Buckley et al. reported about 4% arthrodesis for the ORIF group versus 20% in the conservative group [25]. A reason for our results could again be the higher number of patients with severe fractures as 16 patients (27%) were classified as Sanders type IV and a secondary subtalar arthrodesis was necessary only with these patients.

Many authors agree that treating Sanders types III and IV calcaneal fractures can be challenging and that the calcaneal joint surface is often extensively damaged [27]. Therefore, almost 70% of Sanders type IV comminuted calcaneal fractures can lead to posttraumatic arthritis, while 73% lead to secondary subtalar arthrodesis according to Sanders et al. [13]. Many studies assumed that severe cases with major changes of the Böhler’s angle are at high risk of developing posttraumatic arthrosis and lead to subtalar arthrodesis even with operative fixation and anatomical joint reduction techniques [28].

Lesions and displacement of the peroneal tendon accompanies an intra-articular calcaneal fracture and an anatomical fixation does not guarantee a stable tendon reposition [29].

According to Touissant et al. tendon dislocation often stay undetected. They showed a prevalence of 28% and a significant correlation with Sanders type IV fractures. They suggest furthermore that while ORIF and restoration of the normal anatomy can allow the tendons to be restored to their anatomical position, with the minimally invasive technique, these lesions have a high risk to stay untreated. Nonetheless, is has to be noted that this topic is rarely dealt with in the literature [30].

## Limitations

A major limitation of this study is the absence of a control group treated with a different method and the lack of multicenter randomized controlled studies, which may lead to selection bias. Furthermore, we did not evaluate any peripheral nerve damage, which is common in foot and ankle surgery. Another limitation is the relatively small number of patients and finally the short follow-up time.

## Conclusion

The focus of this study was to show our results of percutaneous minimally invasive external fixation method to treat calcaneal fractures. Our results confirm that a good radiological and functional outcome of DIACFs can be achieved by the 2‑point distractor and that rates of postoperative infections and subsequent arthrodesis were lower than with the traditional ORIF approach described in the literature. The results of this study furthermore confirm that this method is a viable option for severe types of intra-articular calcaneal fractures.

## Data Availability

The datasets used and/or analyzed during the current study are available from the corresponding author upon reasonable request.
